# Stress Assessment of Wild Boar (*Sus scrofa*) in Corral-Style Traps Using Serum Cortisol Levels

**DOI:** 10.3390/ani12213008

**Published:** 2022-11-02

**Authors:** Katharina M. Westhoff, André Fetzer, Kathrin Büttner, Gerhard Schuler, Johannes Lang, Michael Lierz

**Affiliations:** 1 Clinic for Birds, Reptiles, Amphibians and Fish, Faculty of Veterinary Medicine, Justus Liebig University Giessen, 35392 Giessen, Germany; 2 Unit for Biomathematics and Data Processing, Faculty of Veterinary Medicine, Justus Liebig University Giessen, 35392 Giessen, Germany; 3 Clinic of Veterinary Obstetrics, Gynaecology and Andrology, Faculty of Veterinary Medicine, Justus Liebig University Giessen, 35392 Giessen, Germany

**Keywords:** animal welfare, trapping, hunting, glucocorticoids, RIA, SPE, African swine fever

## Abstract

**Simple Summary:**

Corral-style traps for wild boar are used to reduce the number of wild boars. However, many people criticise these traps because of animal welfare issues such as stress and panic. While previous studies focused on behaviour and injuries, this study focused on the so-called stress hormone cortisol. Cortisol levels from trapped animals were compared with levels from animals shot during other hunting methods. Inside traps animals were killed by headshot within 2 h and 17 min after trapping and blood samples were directly taken. Cortisol levels were higher in wild boar killed in traps than in driven hunts and single hunts. Wild boar caught in groups of five or more showed lower cortisol levels than single animals or animals in smaller groups. Therefore, the time animals spend inside the trap and the time of culling all animals should be as short as possible. To reduce stress, it is better to capture larger groups of animals instead of single animals. For the evaluation of harm and stress for wild boar in live traps, cortisol levels alone are not sufficient. Additional information about the behaviour and injuries must be integrated.

**Abstract:**

Capture of wild boar in corral-style traps with subsequent culling is increasingly used for population management. The method is debated due to animal welfare concerns making welfare studies in traps necessary. While previous studies focused on behaviour and injuries, this study dealt with the physiological aspect. Cortisol levels in wild boar caught in corral-style traps (50–90 qm^2^, *n* = 138) were compared with those killed during single (*n* = 37) and driven hunts (*n* = 90). Collected sera were purified by solid phase extraction (SPE) and analysed via radioimmunoassay. Cortisol levels in blood samples were stable under cooled (4–7 °C) conditions for a storage time of up to 87 h before centrifugation. Cortisol levels were significantly higher in wild boar killed in corral-style traps than during driven hunts and single hunts. Wild boar caught in groups of five or more showed lower cortisol levels than single animals or in smaller groups. Therefore, time span inside the trap and of culling should be reduced to a minimum, and capturing groups of animals should be preferred to reduce stress. For animal welfare assessment of wild boar live-trapping, additional data from behavioural analyses and pathological examinations must be integrated.

## 1. Introduction

Wild boar (*Sus scrofa*) population continuously increased during the last decades in Europe [1]. While hunting is the main management tool for wild boar population regulation [2], single hunts (at baiting sites) and driven hunts with hunting dogs are the most common hunting methods used in Germany [3]. Live-trapping of wild boar with subsequent culling has mostly been used as an additional tool for population management in disease prevention and control [4]. With the outbreak and spread of African swine fever in Eastern Europe in 2014, this method received increased attention [5,6,7]. In addition, it is frequently used in areas where low disturbance hunting forms are required, for example, in national parks or in areas where shooting is risky due to security concerns such as in urban areas. However, wild boar trapping is criticised and debated due to animal welfare concerns [8,9]. From an animal welfare and hunting ethics perspective and according to national and international agreements, the condition of the captured animals should be considered when evaluating any trapping method. According to the Agreement on International Humane Trapping Standards (AIHTS [10]) principles, the welfare of the captive animal must be assessed on the basis of physiology, behaviour, and injuries. However, wild boar are not listed as a target species in the AIHTS; these standards are widely distributed regulations for trapping. Additionally, the International Organisation for Standardisation (ISO) developed standards for mammal trapping, but which are not legally binding [11]. Especially, AIHTS are criticised by scientists, who demand new protocols and standards for traps according to the current state of science [12]. While previous studies focused on the behavioural assessment and injuries [13,14], this study focused on the physiological aspect. 

An animal is said to be in a state of stress if it is required to make abnormal or extreme adjustments in its physiology or behaviour in order to cope with adverse aspects of its environment and management [15]. Fried [16] stated that “Methods used to determine if an animal is stressed can be either behavioural or physiological. There is no single measure of stress that can be used in all situations”. Physiologically, an acute stressor leads to the activation of the hypothalamic–pituitary–adrenal (HPA) axis and thus to the release of glucocorticoids. Cortisol is the main active glucocorticoid in pigs, cattle, sheep, mink, fox, and fish [17] and has been used as a stress indicator in various wildlife species and for various stressors such as hunting, handling, trauma, and trapping [18,19,20,21,22,23]. It has also been used for the evaluation of trap-induced stress in wildlife species including wild boar [24,25]. The high variability of cortisol levels poses a challenge for its interpretation [17,22,26]. The HPA axis and thus cortisol levels are affected by diurnal changes, circadian rhythms, and environmental factors and underlie age, sex, and individual variation [27,28,29,30,31]. Previously, the comparison with other traumatic events in order to classify the measured cortisol levels was used [20]. Hamilton and Weeks [22] used shot animals as controls and reasoned that these would best represent the baseline value because of the direct onset of death. 

In principle, cortisol concentrations or their metabolites can be analysed from a variety of sample materials including blood, saliva, faeces, urine, milk, as well as hair and feathers with certain limitations [17,32]. Traditionally, cortisol is measured in blood samples. Evaluating acute stress, blood analysis is best suited [32]. In blood samples, measurably elevated cortisol levels can be observed from about 3 to 5 min after the onset of a stressor, and peak levels are reached within 15–30 min [33]. A negative feedback loop triggered by cortisol causes the return to a baseline level after 60–90 min [32,33]. For the evaluation of cortisol levels in living animals, non-invasive methods are often preferred because handling and restraint lead to the elevation of cortisol levels themselves [34]. Sampling dead animals prevents that, but the sample quality is poorer. There are some challenges in collecting blood samples from shot animals during hunting, especially in driven hunts. For example, taking samples as promptly and cleanly as possible plays an important role, as the coagulation of the blood occurs when blood circulation stops. Additionally, extended storage times may occur, and time of sampling cannot be standardised under field conditions [35]. Haemolytic and lipaemic sera are common findings in samples from shot animals that can lead to problems in analysis [36,37,38]. In particular, haemolytic sera can lead to false results especially when direct steroid immunoassays are used, i.e., without prior adequate sample pre-treatment [39]. Previous studies showed solid phase extraction (SPE) as a valid method for extracting steroids from plasma prior to analysis via immunoassay [40,41]. The cortisol level in serum frozen at −20 °C is stable over years [42], but there is not much information about the pre-centrifugation stability [43]. 

The aim of the study was to analyse cortisol levels in wild boar caught and shot in corral-style traps as part of the welfare assessment of wild boar live trapping. Cortisol levels from animals caught and killed in corral-style traps were compared with animals shot during standard hunting methods (single hunts, driven hunts). Samples from single hunts were included as baseline values for undisturbed animals. Because of various storage times and poor sample quality in shot animals, it was first investigated whether storage time and storage temperature have an influence on the cortisol level in whole blood. 

## 2. Materials and Methods

### 2.1. Study Sites

Wild boar samples were obtained from May 2019 to August 2021 during regular hunting at three sites in Hesse (Central Germany). Site 1 was a state-owned forest (51.0° N/8.8° E), site 2 was an abandoned military site (49.9° N/8.8° E), and site 3 was an active military training area (50.9° N/9.4° E). All three sites consist of mainly mixed broad-leafed forest and therefore named as a prime wild boar habitat in a European context and carry vital wild boar populations, which yielded a hunting bag of >3 wild boars/m^2^ in the last years [44]. Wild boar hunting at all three sites is managed by staff of the responsible forest offices and involves private hunters. At site 1, reference samples from wild boar shot during single hunts were collected from May 2019 to May 2021 and during 15 driven hunts from October to December 2019 and October to December 2020 (Details in Table S1). In all driven hunts, different-sized hunting dogs and beaters were used to find and move animals. Trapping of wild boar took place at all three sites.

### 2.2. Trapping

**Figure 1:**
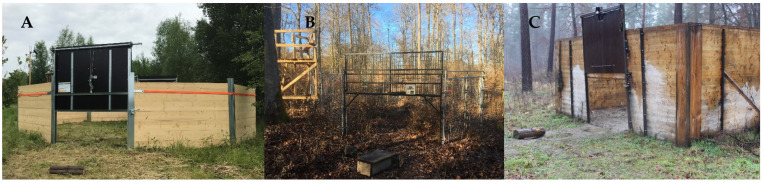
Pictures of the used corral-style traps. (**A**) Krefelder (Krefelder Saufang Vario, Thomas Vennekel & Georg Achten GBR, Krefeld, Germany); (**B**) JagerPro Trap System (Jager Pro^®^, Inc. M.I.N.E.^®^ Trapping System, Columbus, GA, USA); (**C**) Selfmade trap type.

Three different types of corral-style traps were used to capture wild boar. All trap types were mobile with a floor area of 50–90 m^2^ and designed to catch entire family groups. The *Krefelder* trap type (Krefelder Vario, Thomas Vennekel & Georg Achten GBR, Krefeld, Germany) and a *Selfmade* type were made of wooden planks between metal support, and the third trap type *JagerPro* (M.I.N.E.^®^ Trap System, Jager Pro Inc., Fortson GA, USA) was made of mesh wired wall elements (Figure 1). Traps were baited with corn following local restrictions. The closing of the trap was triggered in a controlled manner. A person not on site was informed about entering animals by wildlife camera and triggered the gate via radio or WIFI while watching the animals inside the trap via live video-recording. Wild boars were killed by headshot (rifle with.22 lr calibre) within a mean of 1 h and 32 min (*Max* = 2 h 17 min, *Min* = 39 min) after trapping. In total, 10 traps (*Krefelder*: *n* = 5; *JagerPro*: *n* = 2; *Selfmade*: *n* = 3) were used from October 2019 to August 2021, and 138 wild boars were caught in 27 trapping events (details in Table S2). 

### 2.3. Sampling

Blood samples were collected post mortem in 9 mL sera tubes (S-Monovette^®^, Sarstedt, Nümbrecht, Germany) from the jugular vein or the heart. Samples from 138 trapped animals were directly collected, kept at 4 to 10 °C, and centrifuged within the next 20 h. Hunters collected 53 blood samples from single hunts within a maximum of 2 h 38 min (mean = *M* = 1 h 4 min; standard deviation = *SD* = 40 min; *n* = 51 samples). All sampled animals had a flight distance of <100 m except one, which had a flight distance of 200 m, and information of flight distance was missing in four samples. One sample was collected from an animal that was wounded and tracked afterwards, which was excluded. Blood samples were stored at 4 to 10 °C on site and sent in by hunters via mail to the Clinic for Birds, Reptiles, Amphibians and Fish. The WHO guideline on the use of anticoagulants in diagnostic laboratory investigations states that cortisol is stable in blood at room temperature over 7 days [45]. According to this statement, 16 samples with prolonged storage time (over 7 days) or insufficient sample quality were excluded, and the remaining 37 samples were used for cortisol analysis. A total of 91 samples from driven hunts were collected within a maximum of 5 h 45 min (*M* = 1 h 18 min; *SD* = 1 h 4 min, *n* = 76) and were centrifuged within a maximum of 7 h. Samples from tracked wounded animals (some of which were killed with a knife) were included; only one of the wounded animals was sampled the following day and exceeded the time frames from death to sampling and time until centrifugation, as mentioned before. One sample was excluded due to insufficient sample quality, and the remaining 90 samples were used for cortisol analysis.

All samples were centrifuged at 2000× *g* for 10 min (iFuge D06, Neuation Technologies Pvt. Ltd., Gurajat, India), and sera were frozen at −20 °C immediately until cortisol analysis. Additional information about each sampled animal (single hunt, driven hunt, trapping) was documented on prepared forms. General information was also documented such as date, time, group size, trap type, or hunting type and, for each animal, sex, estimated age category (juveniles, yearlings, over 2 years according to Güldenpfennig et al. [21]), estimated mass, injuries and other abnormalities, number of shots, time of hit shot, sampling, and time of refrigeration and centrifugation of the sample. For animals killed during regular hunting, further information including the behaviour pre- and post-shot, shot distance, and flight distance was recorded on forms handed out to the hunters.

For the pre-study on storage conditions and centrifugation time, a total of 70 blood samples were taken from 10 different wild boars during two trapping events immediately post mortem. One blood sample from each wild boar was directly centrifuged. Three samples each were stored at 4 to 7 °C, and three samples each were stored at room temperature (18–25 °C) for 33 h, 57 h, and 87 h each and centrifuged afterwards as well. After centrifugation, the sera were immediately stored at −20 °C.

### 2.4. Laboratory Analysis—SPE

For the SPE, an extraction system (Avantor^TM^ J.T.Baker^®^ Baker spe-10; Radnor PA, USA) connected to a vacuum pressure pump (KNF Neuberger Laboport GmbH, Freiburg, Germany) with 1 mL disposable extraction columns (Avantor^TM^ BAKERBONDTM spe Octadecyl, C18; Radnor, PA, USA) was used. Frozen sera were thawed and diluted with double-distilled water (1:10) prior to extraction. One millilitre of the diluted sample was centrifuged 2500× *g* for 10 min at room temperature; 2 mL of methanol (≥99.9%, Sigma-Aldrich^®^, Merck KGaA, Darmstadt, Germany) and 2 mL of double-distilled water were applied to the columns with a pressure of 7–8 mm Hg (higher flow rate) for conditioning. Five hundred microlitres of the diluted sample was applied to the column with lower pressure of 2–3 mm Hg. Samples were washed with 2 mL of double-distilled water (under 7–8 Hg mm). SPE columns were dried for approximately 2 min and analytes eluted with 500 µL methanol (95%) at a lower flow rate (2–3 mm Hg). Controls went through identical sample preparation and were derived in each analytical run. Eluates were evaporated to dryness at 49 °C in an infrared vortex evaporator (MicroDancer, Hettich AG, Bäch, Switzerland). The dry extracts were reconstituted in a 1 mL BSA buffer (phosphate-buffered saline pH 7.2/0.1% BSA), and 100 µL was used in radioimmunoassay (RIA).

### 2.5. Laboratory Analysis—RIA

After SPE, the samples were analysed via in-house RIA, as previously published [46,47], applying tritium-labelled tracer and charcoal adsorption for the separation between free and antibody-bound hormone. The anti-serum was directed against cortisol-3-carboxymethyl oxime-BSA. The cross-reactions for androstenedione, 5α-DHT, estradiol-17β, pregnenolone, progesterone, testosterone, and corticosterone were <0.01%. Intra- and interassay coefficients of the variation of the whole measurement procedure including SPE were 4.0% and 7%, respectively; the detection limit was 4 nmol/L. All measurements were performed in duplicate.

### 2.6. Statistical Analysis

All statistics were performed using SAS 9.4 [48]. Cortisol concentrations (dependent variable) of the pre-study were parametric (Shapiro–Wilks test), and an analysis of variance with measurement repetitions with respect to time (t_1_ = 33 h, t_2_ = 57 h, t_3_ = 87 h) and storage temperature (cooled, room temperature) was performed. The t_0_ values were excluded to prevent pseudo replication. 

Cortisol concentrations of the main study (hunting method and group size comparison) were tested for normal distribution beforehand. As they were nonparametric, the data were log10-transformed. One-factor ANOVA was carried out with the log10-transformed data.

## 3. Results

### 3.1. Pre-Study

Analysis of the pre-study data showed a significant influence of storage condition (i.e., temperature) on the cortisol level (*F* (1, 9) = 10.22, *p* = 0.01). Significantly higher cortisol levels were observed under cooled condition than under room temperature (Table 1). Time of centrifugation had no influence on the cortisol level (*F* (2, 8) = 0.13, *p* = 0.88). Interaction between time and temperature had no influence (*F* (2, 8) = 0.42, *p* = 0.67).

### 3.2. Differences between Hunting Methods and Group Size

**Figure 2:**
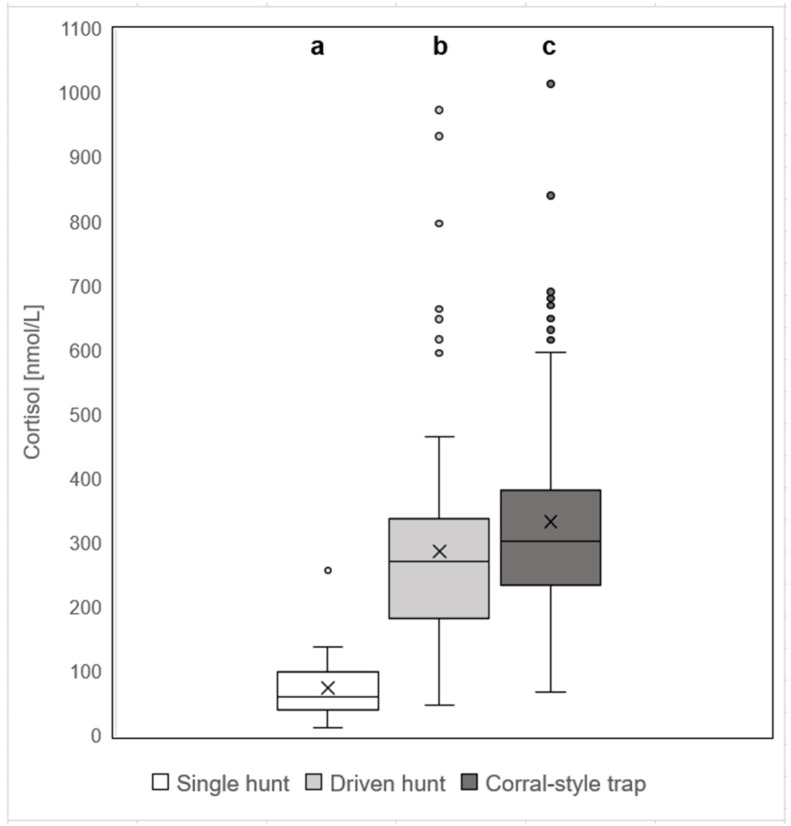
Concentrations of cortisol levels of wild boar shot in single hunts, driven hunts, and trapping. Wild boar caught and shot in corral-style traps (*n* = 138) showed higher cortisol levels than wild boar shot during single hunts (*n* = 37) and driven hunts (*n* = 90). Different superscripts (a, b, c) indicate significant differences in pairwise comparisons (*p* < 0.05).

Comparing the three hunting methods, significant differences in the cortisol levels were observed (*F* = 127.21, *df* = 2, *p* < 0.0001). Wild boar hunted in single hunts (median = *Mdn* = 61.5 nmol/L, *Min* = 12.5 nmol/L, *Max* = 257.6 nmol/L) showed significantly lower cortisol levels than wild boar shot during driven hunts (*Mdn* = 271.9 nmol/L, *Min* = 47.9 nmol/L, *Max* = 973.8 nmol/L) and wild boar shot in corral-style traps (*Mdn* = 302.8 nmol/L, *Min* = 67.3 nmol/L, *Max* = 1014.6 nmol/L), with all differences being significant after Bonferroni correction (single hunt vs. driven hunts and single hunt vs. traps *p* < 0.0001; driven hunts vs. trap *p* = 0.01; Figure 2). In order to exclude any local effect, only trap samples from site 1 were initially included in the statistical evaluation. However, these yielded the same results (Table S3).

**Figure 3:**
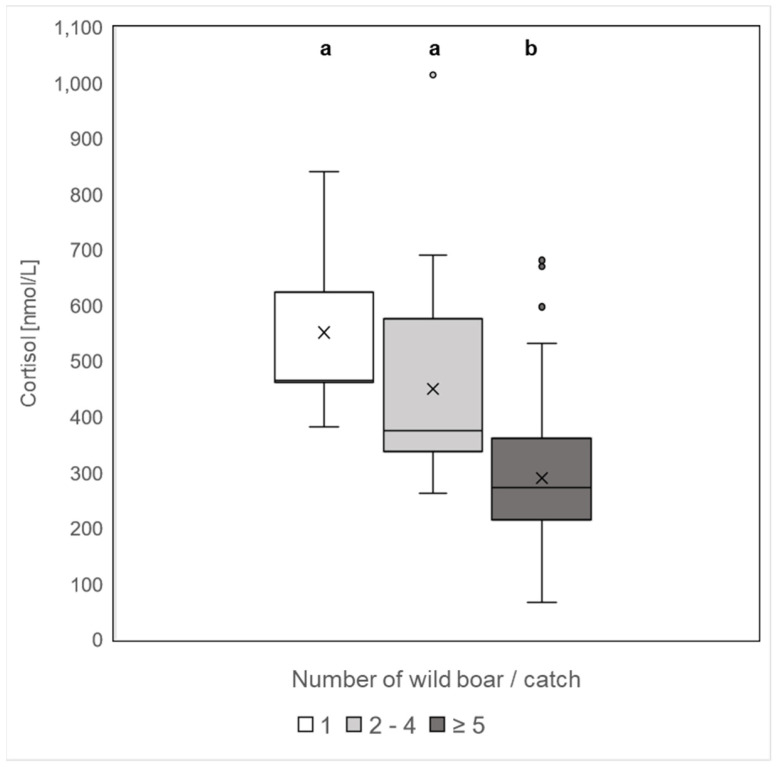
Concentration of cortisol levels of wild boar trapped and shot in corral-style traps of various group sizes. Single wild boar caught and shot in corral-style traps (*n* = 7) showed higher cortisol levels than wild boar in groups of 2–4 (*n* = 26) and in groups greater than or equal to 5 (*n* = 105). Different superscripts (a, b) indicate significant differences in pairwise comparisons (*p* < 0.05).

Cortisol levels according to the group size (1, 2–4, ≥5) of caught wild boar were analysed. Group size had a significant influence on the cortisol level (*F* = 20.77, *df* = 2, *p* < 0.0001). In the pairwise comparison, cortisol levels of wild boar caught in a group of 5 or more (*Mdn* = 272.4 nmol/L, *Min* = 67.3 nmol/L, *Max* = 681.1 nmol/L) were significantly lower than in one (*Mdn* = 465.5 nmol/L, *Min* = 382.4 nmol/L, *Max* = 841.0 nmol/L) or 2–4 (*Mdn* = 375.6 nmol/L, *Min* = 261.9 nmol/L, *Max* = 1014.6 nmol/L) wild boars per catch after Bonferroni correction (1 vs. ≥ 5 and 2–4 vs. ≥ 5 *p* < 0.0001; 1 vs. 2–4 *p* = 0.54; Figure 3).

## 4. Discussion

As part of the animal welfare assessment of wild boar live-trapping, the cortisol levels from wild boar killed in corral-style traps were compared with the cortisol levels of animals shot during driven hunts and single hunts. The advantage of using cortisol for stress analysis is the relative stability of the hormone in blood samples [42,43]. In addition, cortisol is a widely used standard parameter that has been tested in many studies [18,20,22]. Since the cortisol concentration rises first in the blood, analysis from the blood is well-suited for the evaluation of acute stress [32]. The main disadvantage is that the cortisol concentration in the blood is influenced by many factors [27,28,29,30,31]. Therefore, we chose to use reference samples (single hunt and driven hunt), and samples of the single hunts were collected throughout the year to minimise a location and time effect as much as possible.

A disadvantage of using blood samples from shot animals is the sample quality, which severely limits their use for haematology and blood chemistry that has been used in trap evaluation [49]. However, in contrast to blood sampling from live animals, it can be ruled out that stress is triggered through the sampling itself [32].

The present pre-study demonstrated that cortisol levels in blood samples were stable under cooled (4–7 °C) condition for a storage time of up to 87 h before centrifugation. These results emphasise the importance of cooling blood samples during storage until centrifugation prior to cortisol analysis and are contrary to previous results and recommendations [43,45]. The cortisol levels of noncooled blood samples were lower than those of cooled samples. Thus, falsely low levels could occur in blood samples when stored uncooled for a longer time. On the other hand, storage of the blood samples for at least 87 h at 4–7 °C is possible without changes in the cortisol level according to the present study. In the present study, samples from the single hunts were not always continuously cooled due to logistical constraints. The measured cortisol levels may therefore be falsely low according to our results. However, the difference in cortisol levels between cooled and uncooled samples was lower than the difference between hunting methods. Therefore, the difference found in the hunting method comparison is still likely to be significant even if we falsely measured low levels in some samples from single hunts.

Lowest cortisol levels were observed in samples from single hunts. In this hunting method, the hunter observes passing game from greater distance in a high seat. Usually, the game is not exposed to any acute stressor. Ideally, the animal gets killed before it even notices the human or becomes alarmed. Nevertheless, missed shots and wounding can also occur. In this study, we excluded samples of wounded animals in the single hunt data set. In line with these results, Bateson and Bradshaw [38] showed significantly higher cortisol levels of red deer (*Cervus elaphus*) chased by dogs before being shot compared with those of nonhunted deer. Similar results were found in cottontail rabbits (*Sylvilagus floridanus*), where lowest cortisol levels were analysed in shooting compared with trapping and falconry [20]. In order to obtain comparative values, Gentsch et al. [20] considered measurements from "undisturbed animals“ as reference values. We therefore assume that the cortisol levels from single hunts in our study represent basal values and can be used as reference values [20].

A wide scattering of data was found in the cortisol levels from driven hunts. On the average, cortisol levels were higher compared with the levels from single hunts and lower than the levels from trapped animals. This hunting method represents the most inhomogeneous group of all three. Exact shots on undisturbed animals and thus comparable with those from the single hunt can be carried out. However, the game is more in motion due to beaters and dogs, and, thus, a higher number of missed or multiple shots and searches for wounded animals occur. Stressors can, therefore, be very diverse in this hunting method, for example, through chasing and attacking by hunting dogs, wounding by dogs, missed or inaccurate shots, and the presence of humans when chasing. Elevated cortisol levels in red deer and roe deer (*Capreolus capreolus*) have been observed during hunting with dogs and in red deer shot at more than once [20,35,38]. A stress reaction triggered by the human presence has been shown in roe deer during capture in box traps and in wild boar caught in corral-style traps in the form of increased escape behaviour [13,50]. Moreover, wild boar cortisol response is significantly higher under trauma conditions than in reference situations [20].

Furthermore, the time from the occurrence of the stressor to death plays an important role. As mentioned above, serum cortisol is a good indicator of acute stress. Thus, the current stress level is measured via serum cortisol levels. Overlaps of previously experienced stressors make the evaluation of individual levels almost impossible, as the cortisol response can be very complex and does not always decline to the previous level promptly after the peak [51]. The nature and temporal occurrence of stressors experienced by the animals in driven hunts are widely unknown and difficult to detect. Although we used questionnaires to collect data from the hunters about the behaviour of the animals prior to and after the shot, we are mostly unaware of the animals’ experience before getting killed.

Güldenpfennig et al. [21] analysed cortisol in blood samples from wild boar shot during driven hunts and classified 54% as increased trauma levels (>350 nmol/L), 38% as normal cortisol levels (150–350 nmol/L), and 8% out of range according to Gentsch et al. [20]. According to this, 23% of the driven hunt samples from the present study would have to be defined as increased trauma levels, 17% as normal levels, and 50% under 150 nmol/L. The reason for the lower cortisol values in the present study compared with other studies [20,21] might be the different sample preparation and analysis. A general problem in steroid analysis is matrix effects, which can lead to measurement methods that have proven successful for one species or type of sample material being unsuitable or of limited use in other cases. Wudy et al. [39] cautioned against the uncritical use of commercially available test kits on sample material for which they have not been adequately validated due to their potential susceptibility to matrix effects. Accordingly, haemolytic and lipaemic samples, as well as other interfering substances, can pose a problem in the analysis of steroids such as cortisol [37,52]. A common method of eliminating matrix effects is extraction procedures that precede the actual measurement. However, a certain loss of the analyte with an initial extraction method (here SPE) is to be expected [40,41]. Method-specific differences may have the consequence that the measurement results are not readily comparable and that method-specific reference values have to be used for interpretation. Therefore, it is difficult to compare cortisol levels between different studies.

In line with the literature, we found highest cortisol levels in the trapped wild boar. Many articles report that the capture of wild animals leads to increased cortisol levels [18,22]. Since the presence of humans is a stressor for wildlife, as mentioned before, the closure of the gate and the approach of the hunter can be interpreted as two main stressors in wild boar trapping [13]. Gentsch et al. [20] found that “vehicle collisions, diseases, injuries and entanglement combined with post-stress disturbance evoke the greatest stress response of all analysed courses of events”. If wild boars get injured while being trapped, this would consequently lead to the highest cortisol levels after the hunter approaches. Traumatic injuries have been reported in wild boar trapping [13,53]. As trauma leads to increased cortisol levels [20], trap-related injuries will also cause elevated cortisol levels. This could also be a reason for the higher cortisol concentrations compared with single hunts. For animal welfare, capture-related injuries need to be avoided, especially the ones occurring while closing the gate. As trauma length proved to be a decisive factor according to Gentsch et al. [20], the time span in the trap as well as the shooting time should be reduced to a minimum to reduce animal suffering.

Another reason for the elevated cortisol levels compared with the other hunting methods might be the timing of sampling after the stress experience. As mentioned earlier, the peak of the cortisol level is measured 15–30 min after the occurrence of the acute stressor in the blood [33]. Therefore, it is likely that the cortisol levels analysed here represent a value in the range of the peak of the cortisol curve in the blood, since the time of the first shot fell approximately 10–30 min. after the approach of the hunter. It is assumed that the cortisol levels decline in the time between gate closure and shooters’ approach. However, it remains unclear whether they decline to a basal level or to a lower but still elevated level. The latter seems more likely, as various studies have shown that elevated cortisol levels are present in captive animals [18,22].

Casas-Díaz et al. [19] obtained haematologic and biochemical reference intervals for wild boar captured in cage traps. They analysed cortisol levels (*M* = 321.3 ± 195.0 nmol/L) from anaesthetised juvenile and adult wild boars, which were in the range of the ones from the present study [19]. However, it is questionable whether comparing these data is advisable, as they used another analysis method and the animals were sampled under anaesthesia the next morning.

A significant difference in cortisol levels was found when comparing the group size of captured animals. Wild boars in groups of five or more showed lower cortisol levels than single animals or groups of less than five. These findings complement the behavioural observations by Fahlman et al. [13], who found increased escape behaviour in single animals than in animals caught in a group. This is not surprising when dealing with a highly social species. In line with our results, increased plasma cortisol concentrations are reported in domestic pigs when kept isolated [54]. An evaluation of the cortisol levels in relation to age and sex or group composition was not carried out because variation of trapping in the different trap types and sites was too high for an evaluation. Future studies should address these factors in more detail, as age and sex are relevant [21].

## 5. Conclusions

With our results, we provide further information for stress evaluation in wild boar live-trapping and hunting. Wild boar live-trapping with corral-style traps leads to increased cortisol levels compared with standard hunting methods. Therefore, the time span in the trap and time span of shooting should be reduced to a minimum. It is advised to catch large groups instead of single animals to reduce stress. However, cortisol analysis alone is insufficient to assess animal welfare of wild boar corral-style traps as stress is influenced by many factors, and animal welfare should not be based on a single snap by cortisol levels at one time point. For this, additional data from behavioural analyses and pathological examinations need to be integrated, and animal welfare assessment of all different kinds of wild boar live-traps should be investigated. In total, cortisol levels and knowledge to the acute stress situation of wildlife in traps compared with other methods should be used as one tool for welfare evaluation.

## Figures and Tables

**Table 1:** Serum cortisol levels (mean ± standard deviation (nmol/L)) from wild boar (*n* = 10) caught and killed in corral-style traps kept under different storage time and temperature (serum samples *n* = 70; each condition: *n* = 10).

Storage Temperature	Time Point of Centrifugation (Storage Time)				Mean
	t_0_ < 15 min	t_1_ = 33 h	t_2_ = 57 h	t_3_ = 87 h	
Cooled (4–7 °C)	406.4 ± 141.3	387.5 ± 141.6	370.7 ± 176.9	384.6 ± 160.4	380.9 ± 154.8
Room temperature (18–25 °C)		346.2 ± 115.9	341.4 ± 161.7	340.1 ± 158.8	342.6 ± 141.8

## Data Availability

The data presented in this study are available in the supplementary material. Further information or data are available on request from the corresponding author.

## Supplementary Materials

The following supporting information can be downloaded at: https://www.mdpi.com/article/10.3390/ani12213008/s1, Table S1: Date, sex, and estimated age (according to Güldenpfennig et al. [21]) of sampled wild boar from single hunts and driven hunts at site 1 (m = male species; f= female species, j = juvenile; y = yearling; >2 = over 2 years old); Table S2: Number of wild boars caught and killed in trapping events at all sites. Estimated age according to Güldenpfennig et al. [21] (m = male species; f= female species, j = juvenile; y = yearling; >2 = over 2 years old); Table S3: Serum cortisol levels (median, *Min*-*Max* [nmol/L]) from wild boar shot during different hunting methods out of study area 1, test statistics of ANOVA with log10-transformed data (*F* = 106.46, *df* = 2, *p* < 0.0001), and *p* values from post hoc analysis for differences in cortisol levels between hunting methods with Bonferroni correction
