# Supplementary material for: Stress Assessment of Wild Boar (Sus scrofa) in Corral-Style Traps Using Serum Cortisol Levels
Source: Animals (Basel). 2022 Nov 2;12(21):3008. doi: 10.3390/ani12213008 (PMC9654936; doi:10.3390/ani12213008)
Supplement: Supplementary file 1 [file animals-12-03008-s001.zip › animals-1982782-supplementary.pdf]

Supplementary Material

Table S1: Date, sex and estimated age (according to Güldenpfennig et al. [21]) of sampled wild boar from single hunts and driven hunts at site 1 (m = male species; f= female species, j = juvenile; y = yearling; >2 = over 2 years old)

Hunting method	Date	Site	Sex	Estimated age
single	01 May 2019	1	m	y
single	01 May 2019	1	f	y
single	01 May 2019	1	x	y
single	01 May 2019	1	m	y
single	01 May 2019	1	m	y
single	03 May 2019	1	m	>2
single	03 May 2019	1	m	j
single	12 May 2019	1	f	y
single	13 May 2019	1	m	j
single	13 May 2019	1	m	j
single	14 May 2019	1	m	y
single	14 May 2019	1	f	y
single	17 May 2019	1	f	>2
single	19 May 2019	1	m	y
single	24 May 2019	1	m	y
single	09 June 2019	1	m	y
single	10 June 2019	1	m	y
single	04 August 2019	1	f	j
single	16 August 2019	1	m	y
single	17 August 2019	1	f	j
single	30 August 2019	1	f	y
single	03 September 2019	1	f	j
single	18 September 2019	1	m	j
single	18 April 2020	1	f	j
single	23 April 2020	1	m	y
single	24 April 2020	1	m	y
single	24 April 2020	1	m	y
single	29 April 2020	1	f	j
single	08 April 2021	1	m	y
single	11 April 2021	1	m	y
single	21 April 2021	1	m	y
single	24 April 2021	1	f	y
single	28 April 2021	1	m	y
single	29 April 2021	1	m	y
single	08 May 2021	1	f	>2
single	08 May 2021	1	m	j
single	25 May 2021	1	f	j
driven	25 October 2019	1	f	>2
driven	25 October 2019	1	f	>2
driven	25 October 2019	1	m	j
driven	25 October 2019	1	f	j

driven	25 October 2019	1	m	j
driven	25 October 2019	1	m	j
driven	25 October 2019	1	f	>2
driven	25 October 2019	1	f	y
driven	31 October 2019	1	f	>2
driven	31 October 2019	1	m	j
driven	31 October 2019	1	m	j
driven	31 October 2019	1	m	j
driven	31 October 2019	1	f	j
driven	31 October 2019	1	x	x
driven	31 October 2019	1	f	y
driven	09 November 2019	1	f	y
driven	09 November 2019	1	f	y
driven	11 November 2019	1	f	y
driven	11 November 2019	1	f	j
driven	11 November 2019	1	m	j
driven	12 November 2019	1	m	y
driven	12 November 2019	1	m	j
driven	12 November 2019	1	f	j
driven	21 November 2019	1	f	j
driven	21 November 2019	1	m	j
driven	21 November 2019	1	m	j
driven	21 November 2019	1	m	j
driven	21 November 2019	1	f	y
driven	21 November 2019	1	f	y
driven	21 November 2019	1	m	y
driven	21 November 2019	1	f	j
driven	06 December 2019	1	m	j
driven	06 December 2019	1	f	y
driven	06 December 2019	1	m	j
driven	06 December 2019	1	m	j
driven	06 December 2019	1	f	j
driven	06 December 2019	1	m	>2
driven	06 December 2019	1	f	j
driven	06 December 2019	1	m	j
driven	06 December 2019	1	f	y
driven	06 December 2019	1	f	y
driven	06 December 2019	1	m	j
driven	06 December 2019	1	f	j
driven	06 December 2019	1	m	j
driven	06 December 2019	1	f	>2
driven	06 December 2019	1	m	j
driven	06 December 2019	1	m	j
driven	19 November 2020	1	f	y
driven	19 November 2020	1	m	>2
driven	19 November 2020	1	f	j
driven	19 November 2020	1	f	j

driven	19 November 2020	1	f	j
driven	19 November 2020	1	f	j
driven	19 November 2020	1	f	>2
driven	19 November 2020	1	f	j
driven	19 November 2020	1	m	y
driven	19 November 2020	1	f	y
driven	19 November 2020	1	f	y
driven	19 November 2020	1	f	y
driven	20 November 2020	1	f	j
driven	20 November 2020	1	f	j
driven	20 November 2020	1	f	j
driven	20 November 2020	1	m	y
driven	20 November 2020	1	f	j
driven	20 November 2020	1	m	j
driven	20 November 2020	1	f	y
driven	20 November 2020	1	f	y
driven	20 November 2020	1	m	j
driven	20 November 2020	1	f	y
driven	04 December 2020	1	f	j
driven	04 December 2020	1	m	j
driven	04 December 2020	1	f	y
driven	04 December 2020	1	m	j
driven	04 December 2020	1	f	>2
driven	04 December 2020	1	m	j
driven	04 December 2020	1	f	j
driven	04 December 2020	1	f	j
driven	04 December 2020	1	f	y
driven	04 December 2020	1	m	y
driven	04 December 2020	1	f	y
driven	05 December 2020	1	m	j
driven	05 December 2020	1	f	>2
driven	05 December 2020	1	f	j
driven	05 December 2020	1	f	y
driven	05 December 2020	1	f	j
driven	18 December 2020	1	x	j
driven	18 December 2020	1	m	>2
driven	18 December 2020	1	f	>2
driven	18 December 2020	1	f	>2
driven	18 December 2020	1	m	j

Table S2: Number of wild boar caught and killed in trapping events at all sites. Estimated age according to Güldenpfennig et al. [21] (m = male species; f= female species, j = juvenile; y = yearling; >2 = over 2 years old)

No. of trapping event	Date	Site	Trap type	No. of caught and killed animals	Estimated age distribution
1	22 October 2019	1	JagerPro	1	y
2	09 December 2019	1	JagerPro	1	j
3	09 December 2019	1	Eigenbau	6	j
4	15 February 2020	1	JagerPro	5	3 x y, 2 x j
5	05 March 2020	1	Eigenbau	7	j
6	12 March 2020	1	Eigenbau	5	j
7	26 March 2020	2	Eigenbau	4	y
8	14 April 2020	2	Krefelder	3	2 x y, 1 x j
9	15 April 2020	1	Eigenbau	4	j
10	20 June 2020	2	Krefelder	7	2 x y, 5 x j
11	26 June 2020	2	Eigenbau	4	3 x y,1 x j
12	08 July 2020	1	JagerPro	1	j
13	15 July 2020	2	Eigenbau	1	y
14	06 January 2021	2	Eigenbau	7	j
15	17 March 2021	2	Krefelder	2	j
16	08 April 2021	2	Krefelder	10	2 x >2, 8 x j
17	14 April 2021	2	Eigenbau	8	1 x >2, 7 x j
18	25 April 2021	1	JagerPro	1	y
19	03 May 2021	1	Krefelder	20	2 x >2, 2 x y, 16 x j
20	17 May 2021	1	Eigenbau	1	>2
21	26 May 2021	3	Krefelder	3	3 x >2
22	10 June 2021	3	Krefelder	6	1 x y, 5 x f
23	29 June 2021	2	Krefelder	2	2 x y
24	08 July 2021	3	Krefelder	1	>2
25	08 July 2021	2	Eigenbau	15	1 x y, 14 x f
26	06 August 2021	3	Krefelder	9	2 x y, 7 x j
27	14 August 2021	2	Eigenbau	4	4 x >2

Table S3: Serum cortisol levels (median, Min-Max [nmol/L]) from wild boar shot during different hunting methods out of study area 1, test statistics of ANOVA with log10-transformed data (F = 106.46, df = 2, p < 0.0001) and p values from post hoc analysis for differences in cortisol levels between hunting methods with Bonferroni correction

**Serum cortisol concentration Median (*Min-Max*) [nmol/L]**			***p***		
Single hunts (n = 37)	Driven hunts (n = 90)	Trapping (n = 52)	Single hunt vs driven hunt	Single hunt vs trapping	Driven hunt vs trapping
61.5 (12.5-257.6)	271.9 (47.9-973.8)	304.3 (137.1-631.7)	< 0.0001	< 0.0001	0.0217
